# Prevalence and morphology of lower second molars with C-Shaped canals: A CBCT analysis

**DOI:** 10.4317/jced.62368

**Published:** 2025-02-01

**Authors:** Elisabet Mingo, María Noguera, Francisca Jiménez, Maria Llüisa Ballester, Esther Berástegui

**Affiliations:** 1 DDS. Master in Advanced and Experimental Endodontics. Professor of the Master in Advanced and Experimental Endodontics. University of Barcelona, Spain; 2 DDS. Master in Advanced and Experimental Endodontics. University of Barcelona, Spain; 3 PhD, DMD, DDS. Professor of the Master in Advanced and Experimental Endodontics. University of Barcelona, Spain. Researcher of the Idibell Institute; 4 PhD, DMD, DDS. Ex-Director of the Master in Advanced and Experimental Endodontics. University of Barcelona. Researcher of the Idibell Institute

## Abstract

**Background:**

This study aimed to assess the prevalence and morphology of lower second molars with C-shaped configuration among patients at the University of Barcelona Dental Hospital using cone beam computed tomography (CBCT).

**Material and Methods:**

CBCT images of 408 patients, comprising 792 lower second molars, were examined to identify C-shaped canals and evaluate their anatomical characteristics. Inclusion criteria required patients to have both lower second molars present. The configuration of C-shaped canals was categorised at three axial root levels. The variables of symmetry, position, gender and age were analysed.

**Results:**

Out of the 792 lower second molars analysed, a total of 65 molars with C-shaped canals were identified in 42 patients, representing a prevalence of 10.2%. The prevalence in females (12.6%) was significantly higher than in males (6.5%). Bilaterality was observed in 54.76% of individuals with C-shaped molars. The C1 configuration was most frequently observed in the coronal third (56%), while the C2 configuration was equally distributed between the coronal and middle thirds (44.1%). The C3 configuration was most prevalent in the middle third (41.4%), and the C4 configuration was predominantly observed in the apical third (96%). Variations in configurations along the root were observed in 70.8% of the molars. The longitudinal groove was predominantly located on the lingual surface of the roots (83.1%).

**Conclusions:**

CBCT is a useful tool to analyse the morphology of the root canal system.

** Key words:**C-shape, anatomy, prevalence studies, root canal, Cone beam computed tomography, mandibular second molar.

## Introduction

Understanding the internal anatomy of teeth is crucial to perform effective cleaning, disinfection and shaping of the root canal system in a predicTable manner, ultimately enhancing the success rate of endodontic treatments (1).

However, anatomical complexities and variations pose significant challenges for root canal treatment, increasing the risk of procedural accidents and/or persistence of bacteria in missed areas, which can compromise the treatment outcome (2).

Among these root canal system’s variations is the C-shaped canal, so called because of its anatomical cross-sectional conFiguration resembling the letter ‘C’ in both the canal and root (3). Characterized by isthmuses connecting mesial and distal root canals, along with deep chamber floors and fused roots featuring a longitudinal groove, these C-shaped canals can exhibit variations in number and location of root canals, from coronal to apical (1).

C-shaped canals have been widely documented in the literature. In 1991, Melton *et al*. suggested a simplified classification of the different possible cross-sections configurations of these root canal systems (4). In 2004, Fan *et al*. (5) made a modification of this classification, using micro-CT study, which is probably the most widely used in the literature so far (6).

The complex anatomy of C-shaped molars makes the cleaning, shaping and obturation of the root canal system difficult (7). Particularly critical are the apical three millimetres of these molars, where the increased presence of isthmuses makes difficult to achieve complete cleaning and disinfection (1). In addition, the thin dentine wall present in the radicular longitudinal groove elevate the risk of perforation, compromising long-term prognosis (7).

To optimize treatment success, a good knowledge of the C-shaped molars’ anatomy is imperative, coupled with a careful interpretation of their radiographic characteristics (8). Despite the difficulty in identifying them on traditional radiographs, Fan *et al*. propose that the presence of C-shaped molars could be predicted on preoperative radiographs through observation of fused and converging roots (5).

Due to the limitations of diagnosing a C-shaped molar with a 2D radiograph, CBCT emerges as a valuable and non-invasive tool for facilitating accurate diagnosis of these teeth, and for the assessment of morphological characteristics, such as the location of the longitudinal groove (9). While most C-shaped canals are typically observed in lower second molars, they have also been documented in lower first molars, upper first and second molars, and lower premolars (10).

Numerous studies have consistently demonstrated a notable prevalence of C-shaped molars in East Asian countries, ranging from 31% to 45% (1), with a global prevalence of 13.9%. In China, C-molars were identified in 39% of the population (11), while in Korea, they constitute 40% (12).

The prevalence of C-molars is lower in other regions; for instance, a prevalence of 8.9% has been reported in Turkey (13), 3.5% in Brazil (14) and 13% in India (15), among other regions. There is a scarcity of studies analysing the prevalence and characteristics of C-molars in Western countries (16).

Successful endodontic treatment hinges greatly on the precise management of root canal system anatomy (17). Moreover, the C-shaped molar conFiguration stands out as one of the most well-known anatomical variations of the root canal system (6,18,19), presenting a complex anatomy (7,20,21). Early identification of this anatomy, prior to endodontic treatment, facilitates treatment management (22,23). Therefore, the objective of this study was to evaluate the prevalence of C-shaped molars in lower second molars among patients of the University of Barcelona Dental Hospital (HOUB), by analysing CBCT images. Patients’ informed consent for the investigation was obtained prior to CBCT.

## Material and Methods

This study received approval from the HOUB ethics committee with protocol number 37/2024.

Cone beam computed tomography (CBCT) scans were retrieved from the Radiology department database of the HOUB, comprising data from 408 patients who underwent scans. These CBCT scans were conducted for various purposes, including diagnostic and surgical planning for third molar extraction, implant surgery, or as part of orthodontic treatment.

The study’s inclusion criteria stipulated the presence of bilateral lower second molars without prior endodontic treatment. Exclusion criteria encompassed teeth with incomplete root development, root resorption, caries, posts and/or extensive restorations that could impede visualisation.

The radiographic technique employed a kilovoltage of 90kV and 10 mA. All images maintained a maximum voxel size of 0.2 mm and were reconstructed with a slice thickness of 1 mm. The field of view (FOV) size varied from 10x6cm to 10x10cm, encompassing the entire lower arch.

Image analysis was conducted using the Planmeca Romexis® software (Planmeca, Finland). A student operator analysed all collected images, with results subsequently corroborated by an independent evaluator for molars previously identified with C-shaped canals.

**Figure 1:**
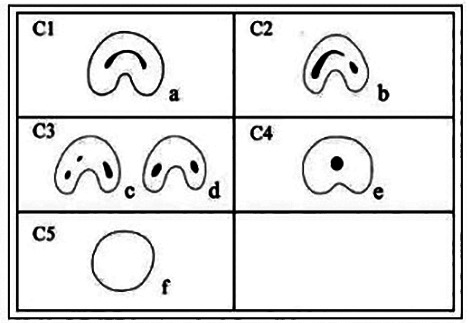
Classification of configurations in molars with C-shaped canals. (a) Type C1; (b) Type C2; (c and d) Type C3; (d) Type C4, and (f) Type C5. Source: Fan *et al*. (5).

The root morphology of all lower second molars underwent evaluation in three planes: axial, coronal, and sagittal, aiming to identify molars with a C-shaped canal. Diagnosis followed the classification proposed by Fan *et al*. (5), which required meeting the following criteria: fused roots, presence of a longitudinal groove, and at least one axial section displaying a C1, C2, or C3 configuration (Fig. 1).

Each identified C-molar was then classified according to its configuration in the axial section at three root levels: coronal, middle and apical. Following the aforementioned classification by Fan *et al*. (5), each configuration was categorized into the following types: C1, representing a continuous C shape; C2, indicating a ‘semicolon’ shape due to a discontinuous C shape; C3, representing two or three separate canals; C4, denoting a single rounded or oval canal; and C5, indicating no canal (Fig. 1).

The position of the longitudinal groove (whether buccal, lingual or both), the molar’s location in the dental arch (whether left or right), the symmetry of the C-shaped molars. as well as the gender and age of the patients were also recorded.

## Results

The collected data underwent statistical analysis using the chi-square test with SPSS for Windows software (SPSS, Chicago, IL), with the significance level set at *P* < 0.05.

Among 872 CBCT images evaluated, 408 met all inclusion criteria, comprising 255 females and 153 males, with an average age of 28 years (range 17-78 years). A total of 791 lower second molars were analysed.

Sixty-five molars with C-shaped canals were identified in 42 patients, accounting for 10.2% of the sample. The prevalence among females was 12.6%, which was significantly higher than that among males (6.5%). Refer to  Table 1 for the distribution of patients with C-shaped second molars by gender.

**Figure 2:**
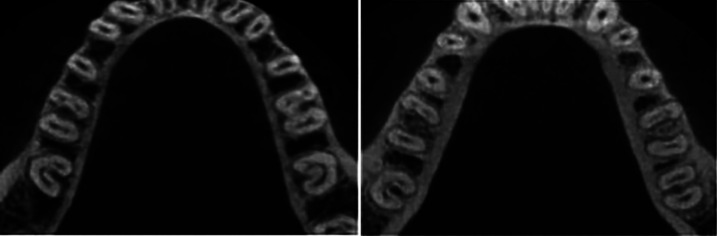
CBCT images of C-shaped molars. (a) Symmetrical and (b) Asymmetrical.

The occurrence of bilaterality in individuals with C-molars was 54.76% (23 patients), while the occurrence of unilaterality was 45.24%, (Fig. 2), with no difference between left or right side.  Table 2 illustrates the distribution of unilaterality and bilaterality, according to gender in patients with C-molars.

**Figure 3:**
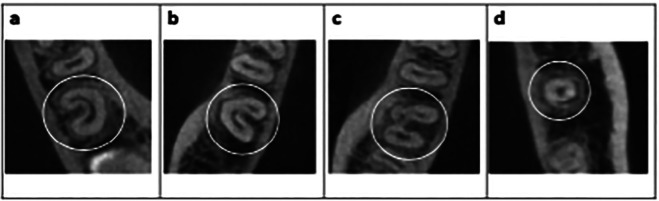
Examples illustrating the classification of the different C-shaped molars configurations, according to the classification by Fan *et al*. (5): (a) Type C1; (b) Type C2; (c) Type C3, and (d) Type C4.

Regarding the type of configurations in the axial section (Fig. 3), the distribution of configurations according to each third can be seen in  Table 3. The C1 configuration was most frequently observed in the coronal third (56%), while the C2 configuration presented the same frequency in the coronal and middle third (44.1%). The C3 configuration was most prevalent in the middle third (41.4%) and the C4 configuration was observed almost entirely in the apical third (96%).

**Figure 4:**
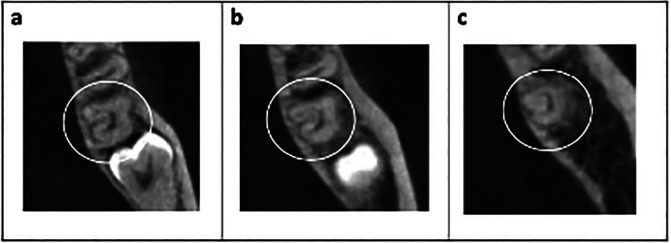
CBCT images of a C-molar with the same C1 configuration: (a) coronal third, (b) middle third, and (c) apical third.

**Figure 5:**
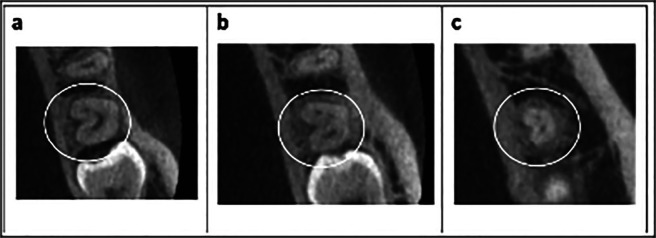
CBCT images of a molar presenting different configurations according to third: (a) C1 in the coronal third, (b) C3a in the middle third, and (c) C3b in the apical third.

It was observed that 29.2% of the C-shaped second molars maintained the same configuration from the coronal to the apical third within the same molar, while 70.8% of the molars exhibited variations in configurations between the root thirds (Figs. 4,5).

**Figure 6:**
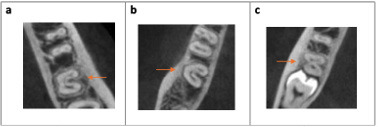
CBCT images of C-shaped molars. The arrows indicate the buccal surface. (a) Buccal longitudinal groove, (b) Lingual longitudinal groove, and (c) Longitudinal grooves on both buccal and lingual walls.

Regarding the location of the longitudinal groove (Fig. 6), the groove located on the lingual surface was the most prevalent with 83.1 %, then 10.8 % on both sides, and 6.2 % on the buccal side.  Table 4 illustrates the distribution of the longitudinal groove according to its location.

**Figure 7:**
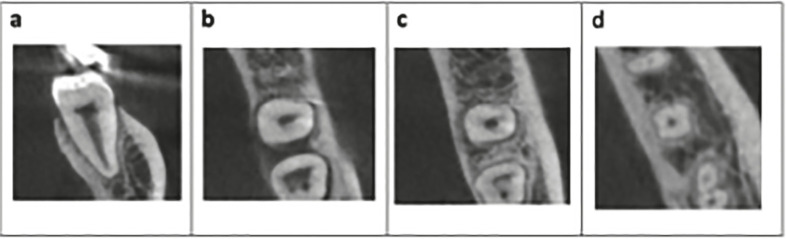
Example of CBCT image of a molar with a single canal. (a) Sagittal section; (b) Coronal, (c) Middle, and (d) Apical third.

Finally, among the 408 patients, 4 were observed to have second molars with single canals (Fig. 7). In total, 7 molars were found across these 4 patients.

## Discussion

Various techniques are available to analyse the morphological characteristics of the root canal system. For the study of C-shaped molars, methods such as diaphanization and staining, periapical radiographs, micro-CT and CBCT scans have been used, among others (24). Diaphanization and micro-CT techniques offer a highly detailed examination of teeth morphology; however, these are techniques that can only be performed on extracted teeth, making them unsuitable for studies involving other variables such as prevalence and tooth position (12,24).

The use of CBCT for studying the morphological characteristics of teeth offers adequate information with a non-invasive approach, eliminating the need for tooth extraction for analysis (18). This technique provides an adequate three-dimensional representation of both the internal and external tooth anatomy, delivering a sufficiently high quality to visualise the morphology of the root canal system. In fact, CBCT can be as specific as diaphanization and staining techniques in identifying the corresponding type of anatomy (11,14,25).

The voxel size used for studying C-shaped molars studies with CBCT typically ranges from 0.125 to 0.250 mm (24). In this study, a voxel size of 2 mm was employed, which has been demonstrated to be adequate for diagnosis and classification of C-molars according to several publications (10,14). Additionally, several C-molar studies have been published using voxel sizes of up to 0.25 mm, achieving satisfactory results for the identification of C-molars (16,18).

Despite the numerous advantages and information provided by CBCT, one aspect to consider in studies using this technique is the requirement for radiation exposure to patients. However, in this study, CBCT images were collected from the database of the University of Barcelona Dental Hospital, where they were indicated for surgical planning or orthodontic treatment design. Consequently, patients were not exposed to unnecessary radiation for this study to obtain information regarding root canal anatomy.

In this study, the diagnosis and classification of C-shaped canals were performed following the criteria proposed by Fan *et al*. (5). According to these criteria, a molar with a C-shaped configuration must fulfil the following characteristics: fused roots, presence of a longitudinal groove on either the lingual or buccal surface of the root, and at least one C1, C2 or C3 configuration in an axial section. It has been described that although the C3 type configurations may appear as two or three independent canals, they are connected between small isthmuses and anastomoses along the root (4). The classification by Fan *et al*. is the most commonly used in studies on prevalence of C-shaped molars by CBCT (7-19), enabling proper comparison between the different studies and populations analysed (24).

It is considered uncommon to find C-configurations other than in lower second molars; however, some cases have been reported in upper and lower first molars, third molars and lower premolars (10). Silva *et al*. (14) reported an incidence of 1.7% of lower first molars with a C conFiguration, and Shemesh *et al*. (9) indicated a prevalence of 0.16%, which is much lower than the prevalence reported for lower second molars, ranging from 3.5% (14) to 44% (16).

Previous studies have demonstrated a high prevalence of C-shaped lower second molars (39-44%) in East Asian countries such as China and Korea (11,12,16), compared to other regions. In this study, the prevalence of C-molars was observed to be 10.2%, which is similar to other studies conducted in Western European countries such as Belgium (10%) (18) and Portugal (8.5%) (24). Von Zuben *et al*. (16) conducted a prevalence study on C-shaped lower second molars using CBCT with standardised protocols across several regions of the world. Spain was one of the countries that participated in this study, where a prevalence of 11% (out of a sample of 400 patients) was obtained. To date, this is the only study that has published prevalence data on C-molar prevalence in this country.

Most prevalence studies using CBCT have been conducted in East Asian countries, but there are publications with varying prevalences from various regions of the world, such as Brazil (3.5%) (14), Chile (10%) (18), South Africa (9.3%) (16), Turkey (8.9%) (13), Russia (14%) (19) and India (13%) (15), among others.

In the study presented here, the individuals analysed only represent a sample of the population attending the University of Barcelona Dental Hospital, and the ethnicity of the patients was not taken into consideration as an inclusion criterion. Determining a specific ethnic group can be technically challenging, as several factors such as a history of migration, a high prevalence of people of multi-ethnic origin and globalisation, among others, must be considered. (18). However, when comparing the prevalence results with studies conducted in other specific populations, it could be said that the sample of this population corresponds to the characteristics of Caucasian groups (19,24).

In this study, the prevalence of C-shaped canals in females was 12.6 %, which was significantly higher compared to males. However, in the literature, it has been described that there is no gender predilection in C-shaped molars (10,11,13). Nonetheless, more recent studies have started to indicate that if there were a difference in prevalence between males and females, it would be statistically significantly higher in females (12,16,24).

C-shaped molars exhibit a complex anatomical configuration, with variations that can appear along the root. In this study, it was observed that the configuration of the C-shaped canals varied in 70.8% of the cases. The C1 and C2 configurations were the most frequent in the coronal and middle thirds, while the C3 configuration presented a similar distribution in the middle and apical thirds, and the C4 configuration had a high prevalence in the apical third. These findings are consistent with those reported by Fan *et al*. (5) and Zheng *et al*. (11).

The prevalence of C1 and C2 types decreased from the coronal to the apical region, whereas C3 and C4 configurations increased towards the apical third. This suggests that the configurations of C1 (described as a continuous ‘C’) and C2 (described as a discontinuous C or ‘semicolon’) have a high probability of subsequently dividing into two or three canals in the middle and apical third (11). Consequently, achieving thorough cleaning and shaping of the canals in the apical region may be challenging in cases of C3 configuration, due to the presence of isthmuses and anastomoses between the canals (12).

Teeth with a C4 configuration (indicating a single canal along the root) are not classified as C-shaped canals according to the criteria set by Fan *et al*. (5). Instead, they have been described as ‘pyramidal’ teeth and have been analysed separately from C-shaped molars, as they are not necessarily related (6). This differentiation is made because these types of teeth, unlike C-configurations, do not present particular challenges in endodontic management (9,19). In this study, 7 second molars with single C-shaped canals were found in 4 patients.

According to the literature, a significant proportion of C-shaped molars exhibit a longitudinal groove on the lingual side (11,12). Martins *et al*. (24) reported that 78% of these grooves are located on the lingual surface. In this study, percentages of 83.1 % on the lingual side, 10.8 % on both lingual and buccal sides, and 6.2 % on the buccal side were observed. The dentin adjacent to this groove is often irregular and thin, which is why this region is commonly referred to as the ‘danger zone’. This area poses a higher risk of perforation and requires appropriate instrumentation techniques (23).

The analysis of symmetry in the identified C-shaped canals revealed a symmetry rate of 54.76%. Literature reports values ranging between 55% (19) and 82% (12), with higher rates typically observed in studies conducted in East Asia (25). Understanding the high percentage of symmetry in C-shaped molars holds clinical significance, as detecting a molar with a C-shaped configuration suggests a high likelihood of finding a similar configuration in the contralateral molar (9,12,24,25).

## Conclusions

The findings of this study underscore the usefulness of CBCT for analysing the morphology of the root canal system. Understanding the complex anatomy and the characteristics of C-shaped molars is essential for accurate diagnosis and effective planning of endodontic treatment.

## Figures and Tables

**Table 1:** Distribution of patients with second molar C according to gender.

	Female	Male	Total
Total patients	255	153	408
Patients with C-molars	32 (12,6%)	10 (6,5%)	42 (10,2%)

**Table 2:** Distribution of C-shaped molars’ symmetry, according to gender.

		Unilateral	Bilateral
Patient	Right	Left	-
Female	9	6	17
Male	2	2	7
Total	11 (26%)	8 (19%)	23 (54.76%)

**Table 3:** Distribution of the different C-shaped molars configurations, by third.

	Coronal	Middle	Apical	Total
C1	28	13	9	50
	56,0%	26,0%	18,0%	100,0%
C2	15	15	4	34
	44,1%	44,1%	11,8%	100,0%
C3	23	36	28	87
	26,4%	41,4%	32,2%	100,0%
C4	0	1	24	25
	0,0%	4,0%	96,0%	100,0%
Total	66	65	65	196

**Table 4:** Distribution of the location of the longitudinal groove of C-shaped molars.

		4,7(n=34)	3,7(n=31)	Total(n=65)
Location:	Lingual	29 (85.3%)	25 (80.6%)	54 (83.1%)
	Buccal	2 (5.9%)	2 (6.5%)	4 (6,2 %)
	Both	3 (8.9%)	4 (12.9%)	7 (10,8 %)

## Data Availability

The datasets used and/or analyzed during the current study are available from the corresponding author.
